# Cell cycle arrest and biochemical changes accompanying cell death in harmful dinoflagellates following exposure to bacterial algicide IRI-160AA

**DOI:** 10.1038/srep45102

**Published:** 2017-03-23

**Authors:** Kaytee L. Pokrzywinski, Charles L. Tilney, Mark E. Warner, Kathryn J. Coyne

**Affiliations:** 1 College of Earth, Ocean, and Environment, University of Delaware, 700 Pilottown Road, Lewes, DE 19958, USA

## Abstract

Bacteria may play a role in regulating harmful algal blooms, but little is known about the biochemical and physiological changes associated with cell death induced by algicidal bacteria. Previous work characterized an algicidal exudate (IRI-160AA) produced by *Shewanella* sp. IRI-160 that is effective against dinoflagellates, while having little to no effect on other phytoplankton species in laboratory culture experiments. The objective of this study was to evaluate biochemical changes associated with cell death and impacts on the cell cycle in three dinoflagellate species (*Prorocentrum minimum, Karlodinium veneficum* and *Gyrodinium instriatum*) after exposure to IRI-160AA. In this study, IRI-160AA induced cell cycle arrest in all dinoflagellates examined. Several indicators for programmed cell death (PCD) that are often observed in phytoplankton in response to a variety of stressors were also evaluated. Cell death was accompanied by significant increases in DNA degradation, intra- and extracellular ROS concentrations and DEVDase (caspase-3 like) protease activity, which have been associated with PCD in other phytoplankton species. Overall, results of this investigation provide strong evidence that treatment with the bacterial algicide, IRI-160AA results in cell cycle arrest and induces biochemical changes consistent with stress-related cell death responses observed in other phytoplankton.

Stressful conditions, including changes in nutrient concentration, light, salinity, culture age and oxidative stress can induce biochemical changes associated with non-necrotic or programmed cell (PCD) death responses in phytoplankton [reviewed in ref. 1]. Among these changes, DNA condensation and degradation^2^,^3^,^4^, the production of reactive oxygen species (ROS)^2^,^5^,^6^,^7^ and increased cysteine-aspartic acid protease-like (caspase-like) activity^4^,^5^,^8^ have been observed in a wide range of phytoplankton species exposed to stressful conditions. Although phytoplankton lack true mammalian caspases they possess proteases that cleave similar sequences. For example, the executioner caspase, caspase-3 recognizes the amino acid sequence DEVD (Aspartate-Glutamate-Valine-Aspartate); therefore the enzymes that cleave the DEVD sequence in phytoplankton are appropriately referred to as caspase 3-like proteases or DEVDases^1^. These markers are considered key elements in PCD responses in phytoplankton [refs 4, 5 and 9, 10, 11, 12 among many others]. Negative interactions with other microbial species may also activate autocatalytic cell death pathways and may play a critical role in population growth and the exchange of nutrients via the microbial loop^13^. Several studies, for example, have investigated the induction of PCD in phytoplankton by viral infection^3^,^14^,^15^,^16^. Although it is thought that bacteria play a role in algal bloom termination^17^, the physiological and biochemical pathways associated with bacterial-mediated cell death in algae have not been investigated.

Pokrzywinski *et al*.^18^ recently demonstrated that the algicidal activity of the bacterium, *Shewanella* sp. IRI-160^19^, was due to a secreted bioactive compound, designated IRI-160AA (where IRI = Indian River Inlet, Delaware, USA, 160 = bacterial isolate #160, AA = algicidal agent). When introduced into culture, the bacterium and/or the bacteria-free filtrate had negative effects on the growth of dinoflagellate species^18^,^19^,^20^,^21^ but did not result in rapid cell lysis. In contrast, there was either no significant effect or a slight stimulatory effect on the growth of other phytoplankton species. The bioactive compound(s) in IRI-160AA was further characterized as a small, hydrophilic, thermally stable molecule(s) with algicidal activity that was most effective when applied during logarithmic growth^18^.

Impacts of IRI-160AA on cellular morphology have also been investigated^22^. Short-term (<2hr) morphological [via super resolution-structured illumination microscopy (SR-SIM)] and ultrastructural [via transmission electron microscopy (TEM)] changes observed in dinoflagellates after treatment with the algicide revealed substantial structural alterations, while the cellular membrane was maintained. Chromosome and nuclear alterations were most apparent, including nuclear and chromosome expulsion and chromosome decompaction in a species-specific manner^20^. These early effects on the dinoflagellate nucleus suggest that the algicide has substantial impacts on the dinoflagellate cell cycle and contributes to the activation of non-necrotic or PCD pathways ultimately leading to cell death.

This and previous studies^18^,^21^,^23^ investing the impacts of IRI-160AA were performed on three dinoflagellate species isolated from the Delaware Inland Bays (Delaware, USA) including *Prorocentrum minimum, Karlodinium veneficum* and *Gyrodinium instriatum*. The three species were chosen as each has a unique feature(s) that can assist with understanding the physiological responses to IRI-160AA and it’s application to dinoflagellates as a group- specific algicide. For example, *P. minimum* is medium in size (18 × 19 μm), is a potentially toxin-producing species and contains cellulosic plates in flattened “amphiesmal” membrane vesicles referred to as a theca. *K. veneficum* (12 × 18 μm) is similar in size to *P. minimum* but is toxic, producing high amounts of Karlotoxin-1 having strong hemolytic properties^24^. *K. veneficum* is also different from *P. minimum* and *G. instriatum* as it possesses a plastid containing fucoxanthin derived from a haptophyte where the other two species, much like the majority of dinoflagellages, contain the pigment peridinin in their plastid^25^,^26^. Furthermore, *G. instriatum* is large (24 × 27 μm), non-toxic and athecate (does not contain membrane bound cellulosic plates). All algal sizes were determined empirically from the laboratory cultures used in this study. The differences in chloroplast structure, surface area to volume ratio, degree of cell covering and toxin status may directly impact the susceptibility of each species to the algicide. For example Tilney *et al*.^21^ found an inverse relationship between algicidal activity and degree of plasma membrane exposure (presence/absence of theca). This suggests that the presence of a theca could delay (or reduce) the entry of the algicide into the cell, though this requires further investigation.

The objectives of this study were to expand our knowledge of the biochemical changes observed in dinoflagellate species in response to IRI-160AA treatment. Results of this study demonstrate that treatment with the algicide altered cell cycle progression and caused an increase in biochemical markers (DNA degradation, reactive oxygen species production and DEVDase activity) typically associated with stress-related cell death pathways in other phytoplankton species.

## Results

### Cell cycle analyses

**Figure 1:**
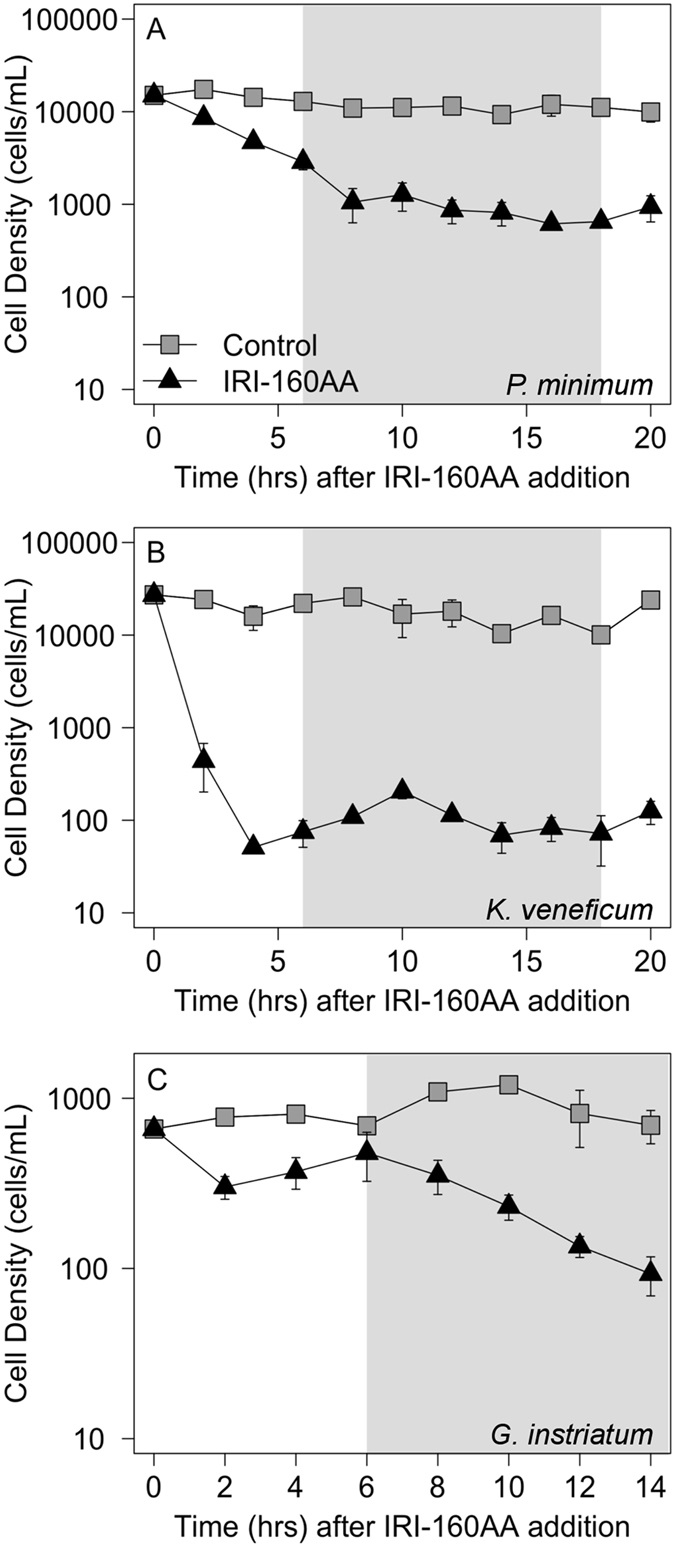
Cell density in dinoflagellate cultures after exposure to IRI-160AA. (**A**) *P. minimum*, (**B**) *K. veneficum* and (**C**) *G. instriatum* after the addition of 10% (*v*/*v) f*/2 (gray squares) or IRI-160AA (black triangles). Points represent the average of triplicates measured every 2 hours for 14 hours (*G. instriatum*) or 22 hours (*P. minimum* and *K. veneficum*). Error bars are one standard deviation of the mean for each point (n = 3). The light gray rectangle identifies the light/dark transition.

*P. minimum* and *K. veneficum* species showed a significant decrease in cell density 2 hours after addition of the algicide (p < 0.05), after which the cell density remained low for the duration of the experiment (Fig. 1A,B). In *G. instriatum*, there was an initial decrease in cell density at 2 hours exposure to IRI-160AA but growth recovered between 4 and 6 hours, prior to the dark phase, after which cell density continually declined for the duration of the experiment (Fig. 1C).

**Figure 2:**
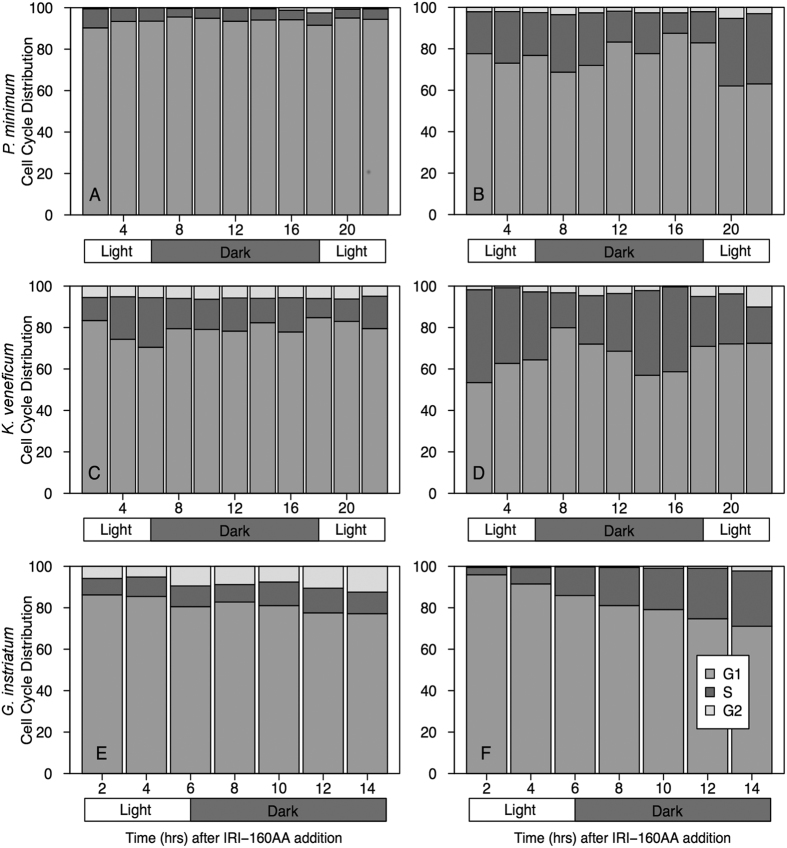
Cell cycle analyses for all three dinoflagellates. The percentage of cells in each phase at each time point for *P. minimum* (**A,B**), *K. veneficum* (**C,D**) and *G. instriatum* (**E,F**) after a 10% (*v*/*v) f*/2 medium (**A,C,E**) or IRI-160AA (**B,D,F**) treatment. Phases were distinguished using the Watson Model and partitioned into G_1_ (gray), S (dark gray), and G_2_/M (light gray). Bars represent concatenated triplicates at 2 hour time intervals. Rectangles under each panel indicate the light/dark transition for reference.

Flow cytometry was used to examine changes in DNA content throughout the cell cycle. All phases of the cell cycle were observed in control cultures (Fig. 2A,C and E). In *P. minimum* control cultures, 5.7% (+/−1.4%) of the population was in S phase for the entire experimental period (Fig. 2A). However, addition of the algicide caused an increase in the number of cells in S to 24% +/− 3.2% (Fig. 2B). Between 12 and 18 hours, just before the lights turned on, the proportion of cells in S dropped to 15% +/−4.0% (Fig. 2B), but then increased to 33% +/− 0.9% for the rest of the experiment. Overall, there was a sizeable increase in cells in S phase with algicide treatment, regardless of exposure time.

In *K. veneficum* control cultures the proportion of cells in S ranged from 9% to 24% of the population (Fig. 2C). In algicide-treated *K. veneficum* the proportion of cells in S phase was higher and more variable than the control, ranging from 17% to 45% of the population (Fig. 2D). From 2 to 8 hours after addition of the algicide, the proportion of *K. veneficum* cells in S declined from 45% to 17%, while from 8 to 16 hrs, the cells in S increased from 23% to 41%. After 16 hrs, the proportion of cells in S remained relatively constant (22% +/− 3.8%) (Fig. 2D). The percentage of cells in S was linked to changes in the light cycle and decreased at each transition (Fig. 2D). In general, there was a considerable increase in the proportion of cells in S phase with IRI-160AA and a concomitant decrease in G_1_.

The proportion of cells in S in control cultures of *G. instriatum* was fairly consistent throughout the experiment ranging from 7.9% to 12% of the population (Fig. 2E). With algicide treatment there was a continuous increase in the proportion of cells in S from 3.6% to 27% of the population over time (Fig. 2F). Overall, there was a substantial increase in S phase with increasing exposure time to IRI-160AA. The *G. instriatum* cell cycle appeared to be independent of light cues.

In summation, cell cycle analysis revealed a decrease of the proportion of cells in G_1_ and an accumulation of cells in S phase in all algicide-treated dinoflagellate cultures. In addition, the proportion of cells in G_2_/M showed non-specific low-level fluctuations between controls and treatments (Fig. 2).

### DNA degradation

**Figure 3:**
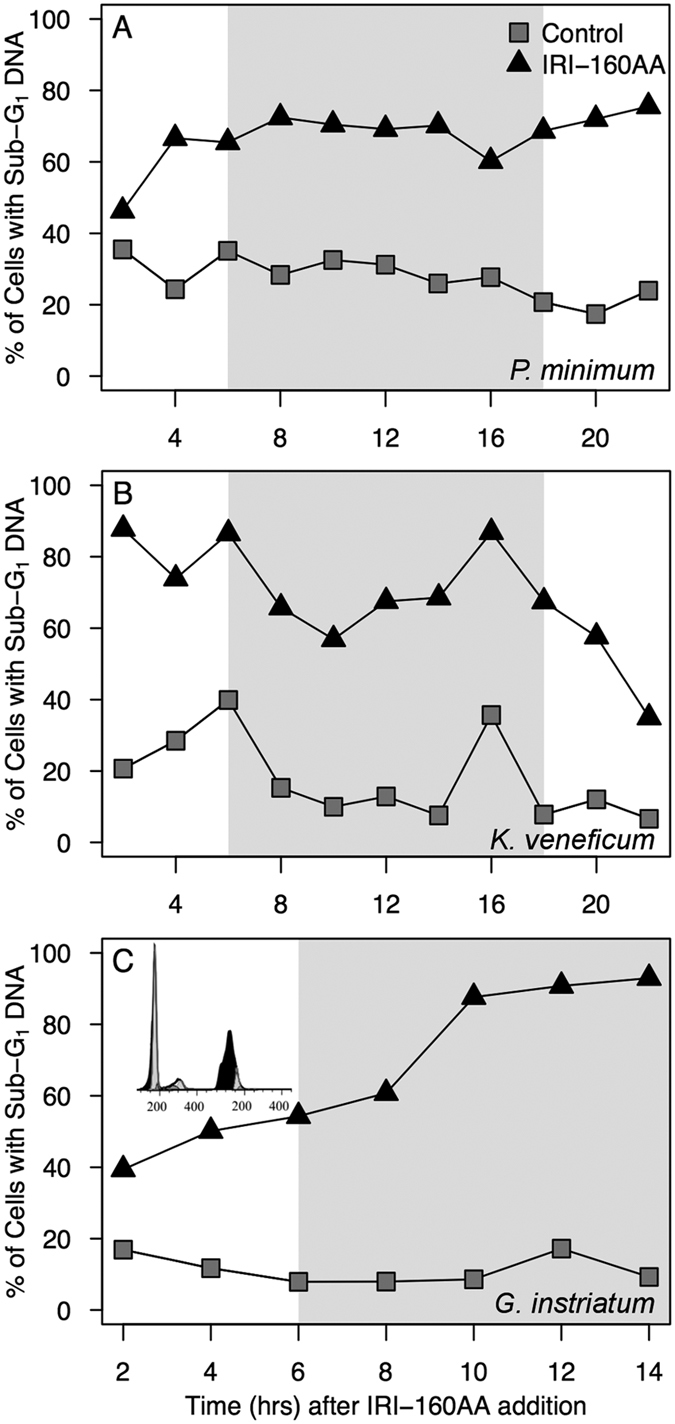
The proportion of *P. minimum* (**A**), *K. veneficum* (**B**) and *G. instriatum* (**C**) cells containing sub G_1_ DNA, which were excluded from the cell cycle analysis. Data points represent concatenated triplicates at 2 hour time intervals of controls (gray squares) and IRI-160AA treatments (black triangles) after a 10% (*v*/*v*) addition to algal cultures. The large gray rectangle indicates the light/dark transition for reference. Panel C, Inset: Representative histograms for concatenated triplicates of *G. instriatum* control (left) and treatment (right) were included to show the proportion of cells with sub G_1_ DNA at 12 hours after algicide addition (in black).

Cells containing less DNA than typically observed in G_1_ are indicated as having “sub G_1_” DNA. Sub G_1_ DNA in control cells may be an artifact of binning, due to the high stringency of the program in identifying cells in G_1_ (see inset, Fig. 3C). In control cultures, the proportion of cells in sub G_1_ remained fairly constant across the experiment (Table S1). In contrast, the proportion of cells with sub G_1_ DNA in algicide- treated cultures varied widely. In algicide-treated *P. minimum*, the proportion of cells with sub G_1_ DNA content increased from 46% at 2 hours to 72% at 4 hours and remained relatively constant throughout the remainder of the experiment (+/−4.3%) (Fig. 3A, black triangles). The proportion of *P. minimum* cells with sub G_1_ DNA was independent of the light cycle. After the addition of IRI-160AA to *K. veneficum*, the proportion of cells with sub G_1_ DNA reached as high as 88% at 2 hours (Fig. 3B). Sub G_1_ DNA-containing cells in the algicide treatments for *K. veneficum* fluctuated, most notably after the lights turned on at 18 hours, where the proportion of cells containing sub G_1_ DNA concentrations decreased to 35% of the population. In *G. instriatum* cultures exposed to the algicide, the proportion of cells containing sub G_1_ DNA increased from 39% at 2 hours, to 93% over the 14 hour exposure (Fig. 3C, black triangles). As with *P. minimum*, the number of *G. instriatum* cells containing sub G_1_ DNA was independent of transitions in the light cycle.

### ROS production

**Figure 4:**
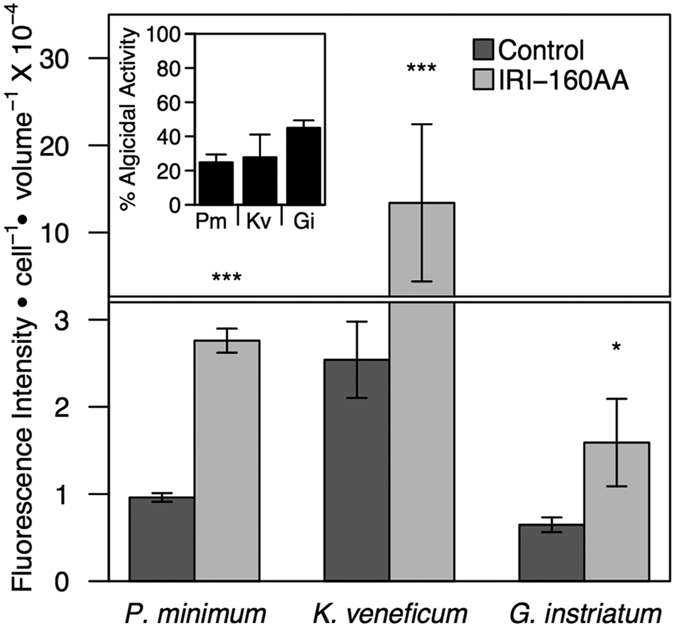
Endogenous ROS production. ROS production after addition of algicide IRI-160AA to *P. minimum, K. veneficum* and *G. instriatum*. Bars represent the mean (n = 3) fluorescence intensity per cell by volume (bio-volume) for controls (dark gray) and algicide treatments (light gray). Error bars represent +/−1 standard deviation. Significant differences between controls and algicide treatments for each species are indicated by asterisks: *p < 0.05; **p < 0.01; ***p < 0.001. The horizontal line represents an axis break. The inset figure represents algicidal activity associated with the endogenous ROS experiment at 3 hours incubation with IRI-160AA.

After 3 hours incubation with the algicide, endogenous ROS was significantly higher in all algicide-treated cultures compared to controls. The highest fluorescence was observed in *K. veneficum* (13.4 × 10^−4^ RFU cell^−1^; p < 0.001), followed by *P. minimum* (2.76 × 10^−4^ RFU cell^−1^; p < 0.001) and *G. instriatum* (1.59 × 10^−4^ RFU cell^−1^; p < 0.05) (Fig. 4). Additionally, there was greater than 20% algicidal activity at 3 hours in all species and up to 45% in *G. instriatum* (Fig. 4 inset).

**Figure 5:**
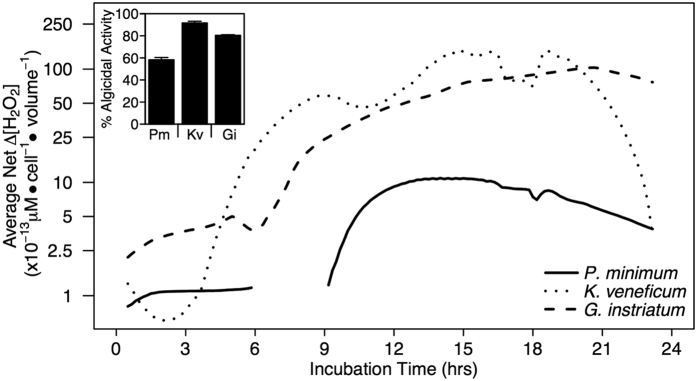
Extracellular production of H_2_O_2_. H_2_O_2_ production after addition of algicide IRI-160AA to *P. minimum* (dashed line), *K. veneficum* (dotted line) and *G. instriatum* (solid line). Points represent the net [average concentration in treatment – average concentration in control; (n = 2)] change in H_2_O_2_ production (μM) per cell, normalized to cell bio-volume. The break in data for *P. minimum* was due to electrical interference. The inset figure represents algicidal activity associated with the H_2_O_2_ experiment where a third replicate was run alongside the H_2_O_2_ sensors. For inset: error bars represent one standard deviation of the mean (n = 3).

Due to differences in species-specific responses to IRI-160AA, experiments to measure extracellular hydrogen peroxide (H_2_O_2_) production were conducted several times for each species over a range of algicide concentrations. Although the magnitude of H_2_O_2_ production varied, these experiments yielded consistent results: addition of the algicide resulted in measurable increases in extracellular H_2_O_2_ (Fig. 5) concurrent with significant levels of algicidal activity (Fig. 5 insert) in dinoflagellate cultures over 23 hours. The data presented here represent the results of one experiment for each species at the algicide concentration that resulted in greater than 30% algicidal activity (Fig. 5 inset). Chl *a* fluorescence was significantly lower in treatments compared to controls for each species, resulting in 58% algicidal activity for *P. minimum* (p < 0.01), 92% for *K. veneficum* (p < 0.01), and 80% activity for *G. instriatum* (p < 0.001). Notable increases in extracellular H_2_O_2_ concentrations were measured in algicide-treated cultures compared to controls, with the highest production observed in both *K. veneficum* and *G. instriatum* and lower concentrations observed in *P. minimum* cultures (Fig. 5; Table S2). In addition, increases in extracellular H_2_O_2_ concentrations (per cell bio-volume) were observed early in *G. instriatum* and *K. veneficum* at 1 and 3 hours, respectively, while increases were not observed until ~9 hours in algicide-treated *P. minimum* (Fig. 5). The peak in extracellular H_2_O_2_ concentration occurred at 15 hours after inoculation for *K. veneficum*, 21 hours for *G. instriatum* and 13 hours for *P. minimum* (Fig. 5). Furthermore, the H_2_O_2_ produced by algicide-treated *G. instriatum* between 16 and 20 hours exceeded the upper limit of detection by the instrument for one of the duplicate H_2_O_2_ sensors.

### DEVDase activity

**Figure 6:**
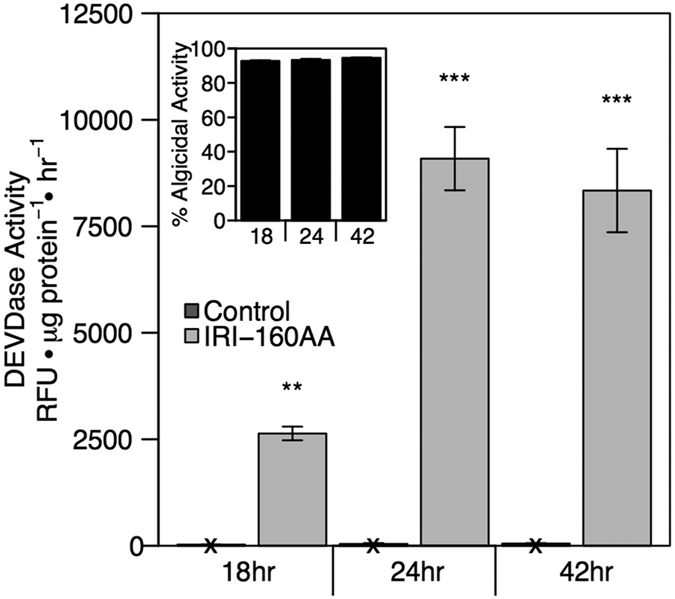
DEVDase enzyme activity: time series. DEVDase activity after addition of algicide IRI-160AA to *G. instriatum* after 18, 24 and 42 hours exposure. Relative activity was normalized to protein content. Bars represent triplicate means of the DEVDase activity expressed as relative fluorescence units per hour per μg of total protein in the control (dark gray) and algicide treatments (light gray). Error bars represent one standard deviation of the mean (n = 3). Significant differences between and treatments are indicated by asterisks: *p < 0.05; **p < 0.01; ***p < 0.001. An X on controls bars indicates little to no DEVDase activity. The inset figure represents algicidal activity accompanying the DEVDase experiment. For inset: error bars show one standard deviation of the mean (n = 3).

Due to differences in species-specific susceptibility to IRI-160AA and differences in the timing of DEVDase activity, several experiments were conducted to determine the optimal concentrations of the algicide and incubation times required for each species for the detection of DEVDase activity without the total degradation of cellular protein. As illustrated in Fig. 6 for *G. instriatum*, algicidal activity increased slightly from 18 to 42 hours (Fig. 6 inset). DEVDase activity in algicide treated cells was significantly higher than controls for all times tested (p < 0.05). Additionally, there was approximately 3 times more DEVDase activity at 24 and 42 hours compared to 18 hours (Fig. 6).

**Figure 7:**
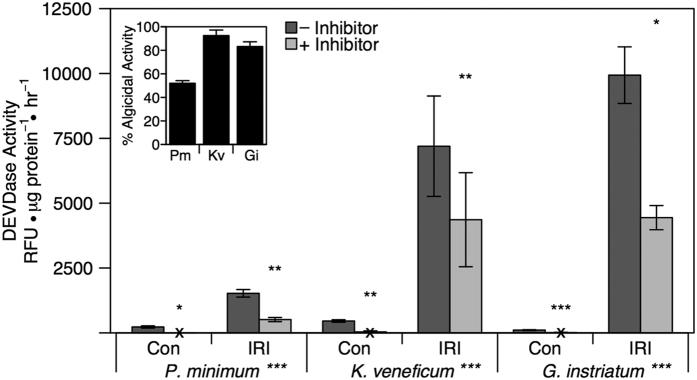
DEVDase enzyme activity. DEVDase activity after addition of algicide IRI-160AA expressed as relative fluorescence units per hour per μg of protein. Activity was measured 18 (*P. minimum*) or 24 (*K. veneficum* and *G. instriatum*) hours after addition of the algicide and normalized to total protein content. Bars represent triplicate means of the DEVDase activity in the presence (light gray) and absence (dark gray) of the Ac-DEVD-CHO inhibitor. Error bars represent one standard deviation of the mean (n = 3). An X indicates little to no DEVDase activity in controls. Significant differences between controls and treatments for each species are next to the species name in the margins while significant differences between + and − inhibitor are above the light gray bars (+inhibitor). In both cases significance is indicated by asterisks: *p < 0.05; **p < 0.01; ***p < 0.001. The inset figure represents algicidal activity accompanying the DEVDase experiment. For inset: error bars represent one standard deviation of the mean (n = 3).

Based on initial experiments, DEVDase activity was then measured in *G. instriatum* at 24 hours, *K. veneficum* at 24 hours and *P. minimum* at 18 hours after algicide addition (Fig. 7). *In vivo* Chl *a* fluorescence was significantly lower in treatments compared to controls for each species during this experiment, resulting in 52% algicidal activity for *P. minimum* (p < 0.001), 93% for *K. veneficum* (p < 0.001), and 83% activity for *G. instriatum* (p < 0.001) (Fig. 7 inset). DEVDase activity was significantly higher in algicide-treated cultures compared to controls in all three dinoflagellate species. DEVDase activity was 6.66-fold (p < 0.001) higher in *P. minimum*, 15.7-fold (p < 0.001) higher in *K. veneficum*, and 91.3-fold (p < 0.001) higher in *G. instriatum* (Fig. 7).

Ac-DEVD-CHO is a Group II specific competitive inhibitor that has a recognition motif preferred by caspases 3, 7, and 2^27^,^28^. Although broad-spectrum FMK inhibitors are commonly used to test for inhibition of caspases, the specificity of Ac-DEVD-CHO for DEVDase activity provided more explicit information about the activity measured in this study^5^,^27^. Addition of the inhibitor Ac-DEVD-CHO decreased the measured DEVDase enzyme activity in both control and algicide-treated cultures (Fig. 7). Although there was little DEVDase activity detected in control cultures, the % inhibition in these cultures were all greater than 89.0% (Fig. 7). There was also a significant decrease (p < 0.05) in DEVDase activity in all algicide-treated cultures when the inhibitor was added, resulting in 36.0% to 66.3% inhibition (Fig. 7).

## Discussion

In this investigation, the impact of IRI-160AA on the dinoflagellate cell cycle as well as biochemical and physiological markers that have been associated with cell death in other phytoplankton species were evaluated in dinoflagellates following exposure to algicide IRI-160AA. Results of this investigation suggest that the algicide inhibits cell cycle progression for all three species tested and induces an autocatalytic, or PCD-like response similar to those as observed in other phytoplankton under stress [refs 5, 7, 9, 10 and 29 among many others]. PCD has been extensively characterized in metazoans^30^ and is an essential process for proper development, function and survival. However, descriptions of PCD have been expanded to include alternative biochemically-mediated death pathways such as those documented in phytoplankton species^1^. These alternative pathways have been classified as apoptosis-like, paraptosis, ferroptosis, aponecrotic or autophagic, but are more generally referred to as PCD^1^,^31^. Although it was not the objective of this investigation to definitively characterize PCD in response to the algicide, the use of approaches similar to those in other studies helps to place this work in a broader context of cell death responses in phytoplankton. For this reason, several markers for PCD were measured, including DNA fragmentation, caspase-like activity and ROS production.

DNA degradation and fragmentation have previously been evaluated through a variety of methods including fluorescence microscopy, gel electrophoresis, TUNEL staining^7^ and cell cycle analysis of cells containing sub G_1_ DNA^32^,^33^. In the current study the proportion of cells containing a full complement of DNA (at or above G_1_ concentrations as determined by flow cytometry) after treatment with algicide IRI-160AA decreased substantially. Although all three species demonstrated substantial DNA degradation with algicide treatment there were inherent species-specific differences in the duration and accumulation of cells containing degraded DNA, likely due to the susceptibility of each species to the algicide. The highest population of cells containing degraded DNA ranged from 69% (24 hour) in *P. minimum*, to 88% (16 hour) in *K. veneficum* and 93% (24hour) in *G. instriatum* (Fig. 3), matching the relative order of sensitivity for these species to the algicide.

Several studies have measured an increase in ROS in phytoplankton in response to a variety of stressors^6^,^7^,^12^,^34^. Ding *et al*.^6^, for example, showed that H_2_O_2_ addition to cultures induced apoptotic-like cell death in *Microcystis aeruginosa* in a dose-dependent manner, resulting in elevated caspase 3-like activity, DNA condensation and fragmentation, decreased efficiency of photosystem II, and increased vacuolization. Additionally, Jauzein and Erdner^29^ showed that the downstream response to thermal stress in the dinoflagellate *Alexandrium tamarense* largely correlated with the degree and duration of ROS production and that a strong and steady increase in ROS reduced photosynthesis and either resulted in the formation of a resting cyst or cell death. In this study, the degree of DNA degradation corresponded directly to the levels of intracellular ROS at 2–3 hours after treatment with IRI-160AA (Fig. 4). Extracellular ROS levels in *K. veneficum* and *G. instriatum* at 18 and 24 hours were also much higher than that of *P. minimum*, consistent with the observed DEVDase activity (Figs 5 and 7). Species-specific differences in the timing and magnitude of ROS production indicate a potential for some species to scavenge excess ROS, perhaps impeding the progression of PCD. Indeed, *P. minimum* contains a novel catalase-peroxidase (KATG) that is known to function in defense against oxidant-induced stress^35^ and may have been activated after exposure to the algicide here, though this was not investigated.

Caspase-like activation has also been observed in a variety of phytoplankton in response to abiotic and biotic stressors. Increases in caspase-like activity have been measured in response to prolonged darkness in the chlorophyte *Dunaliella tertiolecta*^36^, after viral infection of the coccolithophore *Emiliania huxleyi*^14^, during iron starvation in the diatom *Thalassiosira pseudonanna*^9^ and under thermal stress in both the raphidophyte *Heterosigma akashiwo*^37^ and the dinoflagellate *Alexandrium tamarense*^29^, among many others. In the study presented here, DEVDase (caspase 3-like) activity was significantly higher in all three dinoflagellates when exposed to IRI-160AA compared to control cultures. Measurement of increased DEVDase activity and algicidal activity in *G. instriatum* cultures at 18, 24 and 42 hours after inoculation with the algicide confirmed that DEVDase activity coincided with increased cell loss. *G. instriatum* (24 hours) exhibited the highest DEVDase activity, followed by *K. veneficum* (24 hours) and *P. minimum* (18 hours). While activity in *G. instriatum* and *K. veneficum* increased between 18 and 24 hours, DEVDase activity in *P. minimum* decreased after 18 hours, supporting the hypothesis that this species may be better equipped to combat the effects of the algicide. The magnitude of DEVDase activity differed between species but displayed a similar trend as algicidal activity, DNA degradation and ROS production at 18 and 24 hours (*P. minimum* < *K.veneficum* ≈ *G. instriatum*), suggesting that species specific differences may provide a competitive advantage for *P. minimum* in mixed populations in response to IRI-160AA.

For cell cycle analysis, cells containing measurable sub G_1_ DNA levels were first excluded to examine cell cycle progression among cells that retained their full complement of DNA (Fig. 2). There was an increase in the proportion of cells in S phase and a decrease in the proportion of cells in G_1_ in all three species when treated with IRI-160AA compared to controls (Fig. 2). Interestingly, *K. veneficum* and *P. minimum* algicide-treated cells were observed with chromosomes in an anaphase-like arrangement^20^, suggesting that inhibition of the physical separation of chromosomes and the subsequent inability to replicate these chromosomes may be driving cell cycle arrest, potentially resulting in a “pileup” of cells in S phase. However, *K. veneficum* cells may have been able to re-initiate cell cycle progression at later time points (after 16 hours with IRI-160AA) suggesting that, for this species, the algicidal effects on cell cycle progression may be temporary (Fig. 2D).

Whole cells containing measurable sub G_1_ DNA levels were then included to determine if the sub G_1_ population was composed of cells from a specific phase in the cell cycle. If cells in the sub G_1_ population were composed of cells from the G_1_ pool, for example, the proportion of cells in that phase would decrease, as would the proportion of cells in S and G_2_. The S:G_2_/M ratio, however, would remain roughly the same for controls and treatments. Consistent with the cell cycle results discussed above, however, the S:G_2_/M ratio was larger in treatments than in controls at all time points for *G. instriatum* (5–47 times higher than controls) and for all except the last time point for *K. veneficum* (2–42 times higher than controls). Interestingly, the proportion of cells in both S and G_2_/M phases for *P. minimum* was greater in treatments compared to controls (Table S1). Taken together, results of this investigation suggest that exposure to the algicide inhibits progression from S to G_2_/M for *G. instriatum* and *K. veneficum* and through both S and G_2_/M phases for *P. minimum*. It is unclear, however, whether IRI-160AA results in cell cycle arrest through the inhibition of cell cycle checkpoints or by direct interaction with cellular DNA.

The effects of chemical exposure on cell cycle progression in dinoflagellates have been observed in several other studies^38^,^39^,^40^. For example, Leighfield and Van Dolah^39^ examined a phosphodiesterase inhibitor, IBMX to determine if cAMP was involved in cell cycle regulation in *Alexandrium operculatum*. Addition of IBMX to cells in G_1_ resulted in cell cycle arrest in early S-phase^39^. Additionally, Cho *et al*.^41^ evaluated the effect of the metabolic inhibitor 5-fluoro-2′-deoxyuridine on *Alexandrium tamarense* cell cycle regulation and found that addition of the inhibitor also resulted in an accumulation of cells in S phase. However, not all types of stressors induce cell cycle arrest in S. For example, phosphorous depletion caused arrest in G_1_ in several dinoflagellates including *Amphidinium carterae, Karenia mikimotoi, Alexandrium pacificum* and *Prorocentrum donghaiense* (discussed in ref. 42). However, the molecular link between cell cycle regulation, the stress response and programmed cell death remain largely unknown in phytoplankton and it is unclear why certain stressors elicit cell cycle arrest in specific phases. Jauzein and Erdner^29^ demonstrated that with moderate thermal stress, *A. tamerense* cells were able to encyst and become quiescent to avoid cell death. Under high thermal stress, however, the cellular fate was cell death. It was also noted that cell cycle position may influence cellular fate when exposed to abiotic or biotic stressors^29^. In eukaryotes, the G_1_/S transition is regulated by cyclins and their associated kinases (cdks). Homologues of typical eukaryotic cdks and cyclins have been identified in dinoflagellates [discussed in ref. 29] suggesting that dinoflagellate cell cycle progression may be regulated in a similar manner. In mammalian cells, damage to DNA is known to activate cell cycle checkpoints (through cyclins and cdks) and induce growth arrest so that the cell may repair the damaged nucleic acids^43^. Excess ROS may also inflict damage to nucleic acids^44^,^45^,^46^. The rapid surge in ROS from IRI-160AA may have induced nuclear DNA damage, resulting in cell cycle arrest and depending on the degree of cellular damage, programmed cell death.

Bacterial algicides may provide an attractive alternative to other approaches for the short-term mitigation of HABs. Chemical and physical strategies for mitigation of HABs often affect a broad range of species, while algicidal bacteria are capable of controlling a smaller group of target species [discussed in ref. 24]. In addition to specifically targeting only dinoflagellates, the application of IRI-160AA may have a lower environmental impact than other algicidal compounds. For example, bacterial algicides that induce rapid lysis of algal species^17^,^47^,^48^,^49^,^50^,^51^,^52^ have been shown to result in the release of intracellular toxins into the water column^52^, while algicides that induce alternative cell death pathways, such as IRI-160AA, may prevent or reduce the risk of toxin release.

The research described here and from other studies evaluating IRI-160AA^18^,^22^,^23^,^24^,^53^ have shown the potential for inhibiting blooms for a wide range of dinoflagellate species regardless of plastid origin, nutrient acquisition strategy, toxicity, size or degree of plasma membrane exposure without exhibiting significant (<30%) activity against non-dinoflagellate species. However, while the algicide has been effective against all dinoflagellates tested to date some species are more susceptible than others. This and other studies^18^,^21^ have shown that the algicide may be the best at controlling athecate dinoflagellates during logarithmic growth stages and therefore may be best applied during the early stages of a bloom or as a preventive measure in problematic areas. However, further purification and structural elucidation of the active compound(s) need to be conducted before IRI-160AA can be applied in the natural environment.

While the impacts of IRI-160AA on dinoflagellates are promising, the effects on non-target species and higher trophic levels need to be evaluated in more detail. Preliminary studies^20^ on the rainbow trout gill cell line (RTgill-W1, ATCC) showed no cytotoxicity in response to the algicide (up to 20% and 24 hours exposure). Additionally, laboratory microcosms revealed a significant change in the eukaryotic and prokaryotic communities after the addition of IRI-160AA^24^. In total, there was a decrease in dinoflagellate density with a concomitant increase in ciliate, bactivorous chrysophyte and diatom abundances. Collectively this is encouraging for the application of IRI-160AA in mixed natural communities as a management tool for harmful dinoflagellate blooms. However, the application of this or any algicide to control blooms in nature will require extensive research to ensure that collateral damage to higher trophic levels is minimized.

In addition to potential applications of IRI-160AA for controlling dinoflagellate blooms, the algicide could be utilized in basic biochemical studies of dinoflagellates. The algicide may be used as a tool to induce cell death in dinoflagellates, providing a consistent mechanism for the study of cell death pathways in these ecologically relevant organisms. Little is known, for example, about proteases that are upregulated in non-necrotic cell death pathways in dinoflagellates (but see ref. 7) or other protists. Transcriptomic and proteomic approaches would enable the identification of proteases that are differentially expressed during this process. This line of research is of particular interest as it has the potential to challenge conventional views on the evolution of PCD and other non-necrotic cell death pathways in unicellular organisms.

## Material and Methods

### Phytoplankton and bacteria cultures

Cultures of the dinoflagellates *Karlodinium veneficum* (Kareniaceae; CCMP 2936 [National Center for Marine Algae and Microbiota, https://ncma.bigelow.org/]), *Gyrodinium instriatum* (Gymnodiniaceae; CCMP 2935) and *Prorocentrum minimum* (Prorocentraceae; CCMP 2233) were cultured in seawater enriched with nitrate, phosphate, iron (+EDTA), vitamins and trace metals at *f*/2 concentrations (Guillard 1975). Cultures were maintained at 25 °C and at a salinity of 20psu on a 12:12 light:dark cycle at approximately 185 μmol photons m^−2^s^−1^.

*Shewanella* sp. IRI-160 was cultured according to Pokrzywinski *et al*.^18^. Cultures were incubated overnight in liquid LM medium^53^, harvested by centrifugation and washed in 20psu *f*/2 medium. *Shewanella* was then re-suspended in *f*/2 medium and incubated for 1 week at 25 °C. The culture was then centrifuged to remove bacterial cells and autoclaved at 121 °C for 20 minutes, unless otherwise noted.

### Algicidal activity of IRI-160AA

Unless otherwise noted, algal growth was monitored by *in vivo* Chl *a* fluorescence. Pokrzywinski *et al*.^18^ demonstrated that *in vivo* Chl *a* fluorescence could be used to accurately assess changes in cell density after exposure to the algicide. All experiments were conducted on logarithmic phase cultures and *in vivo* Chl *a* measurements of controls and treatments were conducted concurrently for comparison. Algicidal activity was calculated as described in Pokrzywinski *et al*.^18^ using equation (1)^54^,^55^,^56^:


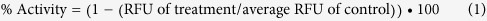


where RFU is the relative fluorescence unit recorded as an *in vivo* Chl *a* fluorescence value. When cell counts were performed, an Olympus BH2 light microscope (OPELCO, Washington D.C., MD, USA) was used with a Neubauer Hemacytometer and algicidal activity was determined as described above.

The number of biological replicates (n) for each experiment ranged from 2 to 4. In cases where an n of 2 was used the sample size was limited by the capabilities of the instrumentation and the constraints of sampling time (described below). The algicide concentration and incubation times were optimized for each species and for each experiment as noted below to produce a response without complete cellular destruction.

### Cell cycle analyses

Cultures of *P. minimum, K. veneficum* and *G. instriatum* were incubated with 10% (*v*/*v*) final concentration of *f*/2 medium (controls) or algicide IRI-160AA in glass culture flasks (n = 3). Algal cell density was monitored using cell counts determined by flow cytometry as described in Marie *et al*.^57^ Cell counts were taken every 2 hours for 14 (*G. instriatum*) or 22 hours (*P. minimum* and *K. veneficum*). Comparisons were made between the starting density (T_0_) and the cell density of controls or treatments at time n (T_n_).

For cell cycle determination, aliquots of each culture were removed every 2 hours for 14 hours (*G. instriatum*) or 22 hours (*K. veneficum* and *P. minimum*) and concentrated by filtering onto polycarbonate filters (TSTP, Isopore membrane, Millipore, Billerica, MA, USA) at ~3 mmHg vacuum (n = 3). Cells were re-suspended and further concentrated by centrifugation at 1500xg for 5 minutes. The supernatant was discarded and pellets were re-suspended in 70% (−20 °C) ethanol to remove chlorophyll and permeabilize the cellular membranes. Cells were mixed by gentle pipetting to avoid clumping and incubated at 4 °C for 30 minutes. Samples were re-centrifuged and re-suspended in fresh cold 70% ethanol for an additional 15 minutes at 4 °C. Cells were concentrated via centrifugation, re-suspended in 750 μL ultra clean DNase free PBS (MO-BIO Laboratories Inc., Carlsbad, CA, USA). After re-suspension, 5 μg RNase A was added and samples were stored at 4 °C until analysis (within 48 hours). Dinoflagellate DNA was stained with Propidium Iodide (PI) at a final concentration of 10 μg/mL. Fluorescence was measured on a FACSCalibur flow cytometer (BD Biosciences, Franklin Lakes, NJ, USA) equipped with a 5 W argon laser with excitation at 488 nm and emission at 585/42 nm. No-stain control and treatment samples were also analyzed to determine Chl *a* extraction efficiency and evaluate PI overlap with Chl *a*.

Cytograms were analyzed with FlowJo version 7.6.5 (Tree Star Inc., Ashland, OR, USA) with the built-in cell cycle analysis package using the Watson (Pragmatic) Model. The Watson Model works by fitting Gaussian peaks to G_1_ and G_2_/M. This model makes no assumptions about the shape of S-phase and fits the data exactly. For all samples the events • s^−1^ did not exceed 1,000. Whole cells were gated using the forward and side scatter cytogram. Doublets (i.e. two cells passing through the flow cell at once) were excluded using the width to area cytogram. Whole cells were gated and cells in G_1_ were identified in the first peak at approximately 200 fluorescence units (FU). Cells in G_2_/M were located in the second peak, approximately twice the distance from G_1_, as G_2_/M cells contain roughly twice as much DNA. Algicide treatments were compared to controls and anything below (to the left of) the G_1_ peak was not included in the Watson model. The proportion of DNA in each phase was determined as a percentage of the total amount of DNA that fit the model and excluded whole cells containing “sub G_1_” DNA contents.

### DNA Degradation

DNA degradation was evaluated at the same time as the cell cycle using the same conditions (n = 3). Intact cells containing lower quantities of DNA than observed in G_1_, identified as sub G1, were indicative of DNA degradation. This portion of the population was excluded from the cell cycle analysis as the sub-G_1_ data did not fit the typical model used for these types of analyses. However, whole cells containing sub-G_1_ DNA were not removed from the initial gating process as they represent a significant portion of the population for algicide treated cells and provide additional information about DNA degradation during cell death.

### Reactive oxygen species (ROS) production

Endogenous ROS production was determined in control and treatment cultures (n = 3) using the carboxy-H_2_DCFDA assay (catalog # C400; Invitrogen/Life Technologies, Grand Island, NY, USA). The algicide was added at 10% (*v*/*v*) final concentration to cultures of *P. minimum, K. veneficum* and *G. instriatum*. Control cultures included *f*/2 medium in place of the algicide at the same final concentration. Endogenous ROS production was measured 3 hours after inoculation with the algicide in 300 μL volumes by addition of 20 μM carboxy-H_2_DCFDA (final concentration). Cultures were incubated for 15 minutes in the dark and fluorescence was measured on a FLUOstar Omega multi-mode microplate reader (BMG Labtech, Cary, NC, USA) with excitation at 485 nm and emission at 520 nm. Control and treatment cultures without added carboxy-H_2_DCFDA were measured to correct for background fluorescence.

Endogenous ROS was calculated as fluorescence per cell by bio-volume. Cell bio-volume was estimated from cell sizes for each species and calculated using equation (2) for an ellipse:


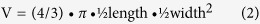


Experiments to measure extracellular ROS (H_2_O_2_) were conducted at room temperature (~23–24 °C) on a 12:12 photoperiod in 30 mL glass scintillation vials with rubber septa. The algicide was added to *P. minimum* at 20%, *K. veneficum* at 10% and *G. instriatum* at 8% final (*v*/*v*) concentration in 10 mL total volume. Controls consisted of *f*/2 medium added at the same concentration. Due to the limited availability of sensors and instrument limitations, controls and treatments were limited to two biological replicates (n = 2). Extracellular H_2_O_2_ production was monitored continuously for 23 hours in each treatment and control culture using 2 mm H_2_O_2_ sensors (World Precision Instruments, Sarasota, FL, USA) with output recorded by TBR4100/1025 Free Radical Analyzer^58^ (World Precision Instruments) as described in Young *et al*.^59^. A running median filter was applied to the data over 2 minute intervals at 0.1 second increments using R version 2.14.0 (The R Foundation for Statistical Computing, Vienna, Austria) and RStudio version 0.97 (RStudio Inc., Boston, MA, USA) to minimize electrical noise. The smoothed medians were then averaged over 10 minute intervals for graphical representation.

The sensors were calibrated at room temperature via amperometeric (electrochemical) determination. The calibration procedure was modified from standard protocols (World Precision Instruments, 2008) as follows: the sensors were allowed to stabilize for 2 hours on 10 nA poise voltage in 0.1 M PBS (0.01 M phosphate buffer, 0.0027 M KCl and 0.137 M NaCl, pH 7.4 at 25 °C). A calibration solution was prepared fresh and consisted of 1.0 mM H_2_O_2_ with ~200 ppm acetanilide as a stabilizer (RICCA Chemical Company, Arlington, TX, USA). To create a calibration curve, the current was measured by consecutively adding increasing amounts of known concentrations of the H_2_O_2_ standard solution and the instrument was allowed to stabilize before the next addition. From this output the calibration curve was generated by plotting the change in current (pA) against the cumulative change in H_2_O_2_ concentration (μM). The concentration of H_2_O_2_ in each control and treatment culture was determined by linear regression analysis and calculated per cell by bio-volume for each species as described for the endogenous ROS production.

### DEVDase activity

The algicide was added to *P. minimum* at 20%, *K. veneficum* at 8% and 16%, and *G. instriatum* at 8% (*v*/*v*) final concentration and control cultures included *f*/2 medium at the same final concentration (n = 3). DEVDase activity was measured at multiple time points for each species (18, 24 and 42 hours for *G. instriatum*; 18 and 24 hours for *K. veneficum* and *P. minimum*) to determine the incubation time with the highest level of activity. DEVDase activity was measured with the Apo-ONE Homogenous Caspase-3/7 Assay (Promega Corp., Madison, WI, USA), according to manufacturer’s protocol. 100 μL of Apo-ONE Caspase-3/7 Reagent was added to 100 μL of control or treatment culture aliquots in triplicate and shaken for 30 minutes in the dark at 300–500 rpm. Fluorescence was measured on a FLUOstar Omega multi-mode microplate reader (BMG Labtech) with an excitation of 485 nm and emission of 520 nm. DEVDase activity was calculated using the measured relative fluorescence unit (RFU) normalized to protein content (described below).

Separate reactions included the reversible caspase inhibitor, Ac-DEVD-CHO (Promega Corp., Madison, WI, USA), to assess the specificity of DEVDase activity in algal cells. The concentration of inhibitor used in this study was determined empirically with initial concentrations as per manufacturer’s suggestions. The assay was performed as described above with the addition of 10 μM Ac-DEVD-CHO to each treatment and control reaction before the addition of the Apo-ONE Caspase-3/7 reagent. DEVDase activity in the presence of the inhibitor was calculated as above, and normalized to protein content. Percent inhibition was calculated using equation (3):


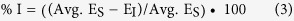


Where E_I_ and E_S_ represent fluorescence in the presence and absence of the inhibitor, respectively.

Cells were harvested for total protein analysis by centrifugation. The cell pellets were re-suspended in 200 mM KPi buffer (27.2 gL^−1^ KH2PO4, 8.0 gL^−1^ KOH, pH 7.9) and sonicated at 20 kHz at an output level of 2.0 using a microtip on a Sonicator 3000 (MISONIX Inc./QSonica, LLC. Newtown, CT, USA) for 60 seconds with 5 second pulses to avoid overheating. The supernatant was frozen at −80 °C until quantification using the Pierce BCA and Micro BCA Protein Assay Kits (Thermo Scientific, Rockford, IL, USA).

### Statistical analysis

Statistical significance was calculated using R version 2.14.0 and R-Studio version 0.97. For the algicidal activity, analyses were performed using a one-way ANOVA to compare changes in cell densities (based cell counts or on *in vivo* Chl *a* fluorescence) of controls and treatments for each of the three species. In the cell cycle experiment the significance of differences in cell counts between controls and algicide treatments were assessed using a two-way repeated measures ANOVA with treatment and time as the factors. For cell cycle determination and DNA degradation experiments, no statistical analyses were performed as analyses were conducted on concatenated triplicates in order to increase the number of cells in the algicide treatments. For intracellular ROS experiments, comparisons were made using a one-way ANOVA between control and treatment fluorescence intensities for each species. No statistical analyses were conducted for extracellular H_2_O_2_ production due to the low sample size (n = 2) as a result of instrument limitations. For DEVDase activity a one-way ANOVA was used to calculate statistical differences between controls and treatments. A paired *t*-test was then used to compare the inhibited and un-inhibited normalized DEVDase enzyme activities for controls and treatments, where controls and treatments were analyzed separately. Where applicable (one or two-way ANOVAs), p-values were adjusted for independent species using a post-hoc Tukey’s test to account for multiple comparisons. In all cases an alpha level of 0.05 was considered significant.

## Additional Information

**How to cite this article:** Pokrzywinski, K. L. *et al*. Cell cycle arrest and biochemical changes accompanying cell death in harmful dinoflagellates following exposure to bacterial algicide IRI-160AA. *Sci. Rep.* **7**, 45102; doi: 10.1038/srep45102 (2017).

**Publisher's note:** Springer Nature remains neutral with regard to jurisdictional claims in published maps and institutional affiliations.

## Supplementary Material

Supplementary Table S1 and S2
