# Supplementary material for: Cell cycle arrest and biochemical changes accompanying cell death in harmful dinoflagellates following exposure to bacterial algicide IRI-160AA
Source: Sci Rep. 2017 Mar 23;7:45102. doi: 10.1038/srep45102 (PMC5362807; doi:10.1038/srep45102)
Supplement: Supplementary Table S1 and S2 [file srep45102-s1.doc]

|  | Treatments | |  |  | Controls |  |  |  |
| --- | --- | --- | --- | --- | --- | --- | --- | --- |
| Time point | G_1_ | S | G_2_ | Excluded | G_1_ | S | G_2_ | Excluded |
| *P. minimum* |  |  |  |  |  |  |  |  |
| 2 | 0.4169 | 0.1090 | 0.0113 | 0.4628 | 0.5824 | 0.0596 | 0.0032 | 0.3548 |
| 4 | 0.2439 | 0.0831 | 0.0068 | 0.6661 | 0.7066 | 0.0487 | 0.0015 | 0.2432 |
| 6 | 0.2652 | 0.0713 | 0.0088 | 0.6546 | 0.6070 | 0.0414 | 0.0007 | 0.3509 |
| 8 | 0.1894 | 0.0765 | 0.0097 | 0.7244 | 0.6844 | 0.0291 | 0.0031 | 0.2834 |
| 10 | 0.2128 | 0.0751 | 0.0078 | 0.7044 | 0.6405 | 0.0331 | 0.0012 | 0.3252 |
| 12 | 0.2567 | 0.0459 | 0.0057 | 0.6916 | 0.6430 | 0.0435 | 0.0015 | 0.3120 |
| 14 | 0.2316 | 0.0588 | 0.0078 | 0.7017 | 0.6967 | 0.0404 | 0.0035 | 0.2593 |
| 16 | 0.3484 | 0.0393 | 0.0105 | 0.6018 | 0.6811 | 0.0331 | 0.0087 | 0.2770 |
| 18 | 0.2598 | 0.0471 | 0.0066 | 0.6865 | 0.7258 | 0.0471 | 0.0200 | 0.2071 |
| 20 | 0.1739 | 0.0915 | 0.0149 | 0.7198 | 0.7852 | 0.0353 | 0.0057 | 0.1738 |
| 22 | 0.1543 | 0.0829 | 0.0075 | 0.7554 | 0.7183 | 0.0385 | 0.0040 | 0.2393 |
| *K. veneficum* |  |  |  |  |  |  |  |  |
| 2 | 0.0652 | 0.0547 | 0.0021 | 0.8780 | 0.6613 | 0.0883 | 0.0437 | 0.2067 |
| 4 | 0.1637 | 0.0953 | 0.0021 | 0.7389 | 0.5316 | 0.1469 | 0.0368 | 0.2847 |
| 6 | 0.0868 | 0.0442 | 0.0038 | 0.8652 | 0.4236 | 0.1440 | 0.0336 | 0.3988 |
| 8 | 0.2736 | 0.0578 | 0.0110 | 0.6577 | 0.6730 | 0.1239 | 0.0503 | 0.1528 |
| 10 | 0.3106 | 0.1007 | 0.0200 | 0.5688 | 0.7118 | 0.1313 | 0.0571 | 0.0998 |
| 12 | 0.2226 | 0.0904 | 0.0116 | 0.6753 | 0.6819 | 0.1395 | 0.0500 | 0.1287 |
| 14 | 0.1791 | 0.1285 | 0.0068 | 0.6855 | 0.7610 | 0.1088 | 0.0546 | 0.0756 |
| 16 | 0.0770 | 0.0538 | 0.0004 | 0.8688 | 0.5008 | 0.1065 | 0.0360 | 0.3567 |
| 18 | 0.2307 | 0.0783 | 0.0163 | 0.6747 | 0.7816 | 0.0854 | 0.0551 | 0.0779 |
| 20 | 0.3055 | 0.1023 | 0.0159 | 0.5763 | 0.7296 | 0.0952 | 0.0548 | 0.1204 |
| 22 | 0.4708 | 0.1138 | 0.0656 | 0.3498 | 0.7424 | 0.1459 | 0.0457 | 0.0660 |
| *G. instriatum* |  |  |  |  |  |  |  |  |
| 2 | 0.5813 | 0.0217 | 0.0031 | 0.3939 | 0.7165 | 0.0658 | 0.0487 | 0.1690 |
| 4 | 0.4563 | 0.0393 | 0.0031 | 0.5013 | 0.7543 | 0.0830 | 0.0456 | 0.1171 |
| 6 | 0.3928 | 0.0631 | 0.0012 | 0.5428 | 0.7416 | 0.0922 | 0.0872 | 0.0790 |
| 8 | 0.3183 | 0.0721 | 0.0022 | 0.6074 | 0.7616 | 0.0779 | 0.0808 | 0.0796 |
| 10 | 0.0978 | 0.0246 | 0.0011 | 0.8764 | 0.7407 | 0.1040 | 0.0693 | 0.0860 |
| 12 | 0.0690 | 0.0225 | 0.0009 | 0.9077 | 0.6419 | 0.0987 | 0.0873 | 0.1720 |
| 14 | 0.0500 | 0.0188 | 0.0016 | 0.9297 | 0.7000 | 0.0944 | 0.1130 | 0.0926 |

**Cell Cycle Arrest and Biochemical Changes Accompanying Cell Death in Harmful Dinoflagellates Following Exposure to Bacterial Algicide IRI-160AA**

Kaytee L. Pokrzywinski, Charles L. Tilney, Mark E Warner & Kathryn J. Coyne

Table S1. Proportion of cells in each phase of cell cycle, G_1_, S and G_2_, and proportion of cells excluded due to less than G_1_ (sub G_1_) DNA contents.

Table S2. Net extracellular change in H_2_O_2_ concentration (μM per cell by bio-volume) after treatment with IRI-160AA, broken down by replicate. This table shows each replicate in the average for Fig 5. No control exceeded an H_2_O_2_ concentration of 2x10^-15^ μM per cell by bio-volume at any time point evaluated. NA indicates electrical interference. The gray block represents when the H_2_O_2_ concentration exceeded the capabilities of the instrumentation.

|  | *P. minimum* | | *K. veneficum* | | *G. instriatum* | |
| --- | --- | --- | --- | --- | --- | --- |
| Time (hrs) | Rep 1 | Rep 2 | Rep 1 | Rep 2 | Rep 1 | Rep 2 |
| 1 | 1.82E-13 | 4.20E-15 | 0 | 1.86E-13 | 4.27E-13 | 8.31E-14 |
| 2 | 2.14E-13 | 7.12E-16 | 0 | 1.26E-13 | 5.53E-13 | 9.13E-14 |
| 3 | 2.22E-13 | NA | 0 | 1.55E-13 | 6.35E-13 | 9.74E-14 |
| 4 | 2.23E-13 | NA | 1.37E-13 | 3.11E-13 | 7.07E-13 | 1.04E-13 |
| 5 | 2.28E-13 | NA | 8.73E-13 | 8.02E-13 | 8.29E-13 | 1.04E-13 |
| 6 | 1.57E-13 | NA | 1.95E-12 | 2.09E-12 | 7.53E-13 | 6.39E-14 |
| 7 | 1.96E-15 | NA | 2.78E-12 | 4.12E-12 | 1.20E-12 | 6.08E-14 |
| 8 | 1.11E-14 | NA | 3.91E-12 | 6.01E-12 | 2.81E-12 | 8.25E-14 |
| 9 | 2.08E-13 | NA | 4.40E-12 | 7.16E-12 | 4.45E-12 | 1.18E-13 |
| 10 | 6.64E-13 | 7.83E-14 | 3.42E-12 | 6.82E-12 | 5.84E-12 | 1.95E-13 |
| 11 | 1.09E-12 | 2.96E-13 | 2.32E-12 | 7.23E-12 | 7.34E-12 | 2.88E-13 |
| 12 | 1.33E-12 | 4.93E-13 | 2.02E-12 | 9.96E-12 | 8.85E-12 | 3.76E-13 |
| 13 | 1.46E-12 | 6.24E-13 | 2.41E-12 | 1.47E-11 | 1.02E-11 | 4.61E-13 |
| 14 | 1.45E-12 | 7.04E-13 | 4.12E-12 | 2.03E-11 | 1.17E-11 | 6.18E-13 |
| 15 | 1.40E-12 | 7.56E-13 | 6.14E-12 | 2.20E-11 | 1.36E-11 | 9.92E-13 |
| 16 | 1.28E-12 | 7.75E-13 | 6.65E-12 | 2.07E-11 | 1.43E-11 | 1.56E-12 |
| 17 | 1.16E-12 | 6.39E-13 | 4.66E-12 | 1.31E-11 | 1.43E-11 | 2.31E-12 |
| 18 | 1.00E-12 | 6.19E-13 | 6.62E-12 | 1.21E-11 | 1.43E-11 | 3.32E-12 |
| 19 | 9.37E-13 | 6.36E-13 | 1.04E-11 | 1.62E-11 | 1.43E-11 | 4.42E-12 |
| 20 | 8.68E-13 | 4.60E-13 | 5.74E-12 | 1.45E-11 | 1.43E-11 | 5.65E-12 |
| 21 | 7.67E-13 | 3.68E-13 | 1.36E-12 | 1.06E-11 | 1.40E-11 | 6.14E-12 |
| 22 | 6.69E-13 | 2.93E-13 | 0 | 6.67E-12 | 1.28E-11 | 5.39E-12 |
| 23 | 5.82E-13 | 2.30E-13 | 0 | 3.60E-12 | 1.12E-11 | 4.88E-12 |
